# Environmental Health Research and the Observer’s Dilemma

**DOI:** 10.1289/ehp.0900861

**Published:** 2009-05-26

**Authors:** David B. Resnik

**Affiliations:** National Institute of Environmental Health Sciences, National Institutes of Health, Department of Health and Human Services, Research Triangle Park, North Carolina, USA

**Keywords:** beneficence, environmental health research, ethics, human subjects, observation, regulations, risk, risk communication

## Abstract

**Background:**

Environmental health researchers frequently study people in occupational, educational, recreational, or domestic settings who are exposed to hazardous agents.

**Objective/discussion:**

Deciding whether—and how—to inform research subjects about risks they face in their environment can be a challenging task for investigators. Because legal rules and professional guidelines do not cover this topic, investigators must carefully consider their ethical obligations in light of the facts and circumstances.

**Conclusion:**

To navigate through this dilemma, investigators should consider the evidence for the risks, the nature of the risks, the usefulness of risk information to the subjects, and the effects on the study and community of informing subjects about risks.

Environmental health researchers frequently study people in occupational, educational, recreational, or domestic settings who may be exposed to hazardous agents, such as pesticides, heavy metals, toxic chemicals, allergens, and tobacco smoke (Chiang et al. 2008; Lu et al. 2008; Ma et al. 2008; Sheehan et al. 2009; Theis et al. 2008). The knowledge gained from this research can improve our understanding of how environmental agents affect human beings, and can be useful in making decisions aimed at preventing diseases or promoting health. For example, parents can use information about how children are exposed to pesticides to reduce their children’s exposures. Government agencies can also use this information to develop and implement pesticide regulations to protect the children from harmful exposures (Cranor 1993).

Observing people who are exposed to hazards in their environment can create ethical dilemmas for investigators (Resnik and Wing 2007; Resnik and Zeldin 2008). Consider the following hypothetical scenario: Researchers are planning to study the effects of dichlorodiphenyltrichloroethane (DDT) on pregnancy loss in females living in a sub-Saharan country in which DDT is sprayed indoors to control mosquitoes that carry malaria. The investigators do not know that DDT exposure increases the risk of pregnancy loss, but evidence from animal studies suggests that it might, and they want to learn more about the effects of DDT on pregnancy. The investigators will take blood samples, measure DDT levels in the homes, interview subjects, and collect health information. In the 1970s, the United States and many other developed countries banned the use of DDT because of its toxic effects on wildlife [U.S. Environmental Protection Agency (EPA) 2007]. Although DDT’s effects on human beings have not been thoroughly investigated, there is some evidence that DDT may have adverse teratogenic effects (Kegley et al. 2008), and DDT is reasonably anticipated to be a human carcinogen (National Toxicology Program 1985).

## The Observer’s Dilemma

Should the investigators tell the research subjects about the potential risks of DDT exposure? If so, what should they tell them? The information that they share with research subjects could encourage them to reduce their exposure to DDT, which may affect the results of the study. Also, if the villagers become concerned about the health risks of DDT, they may decide to stop using it to kill mosquitoes, which could hamper efforts to control malaria. The basic dilemma that the investigators face, which I will call the observer’s dilemma, involves deciding whether to remain a neutral observer when doing fieldwork or to take some steps to protect the people you are studying from harm. Other disciplines that engage in fieldwork, such as anthropology, also deal with this dilemma. For example, an anthropologist who observes a murder must decide whether to report the crime or remain silent (Cassell and Jacobs 2006).

To understand the nature of this dilemma, it will be useful to distinguish between risks directly related to and not directly related to research (Wendler and Miller 2007). A risk directly related to research is one that occurs as a result of participating in a study, such as a risk associated with a procedure or method. A risk not directly related to research is one a person would face even if he or she is not participating in research. For example, if a person works as a pesticide applicator for an industrial farm, he encounters risks associated with pesticide exposures. If an investigator collects data on pesticide applicators, the risk of applying pesticides is a risk not directly related to research, because the applicators would face this risk even they did not participate in the study. Risks associated with procedures the investigator uses to collect data, such as the risks of interviews or blood collection, would be risks directly related to research. In the DDT case mentioned above, the villagers are already using DDT to control malaria-carrying mosquitoes. The research study does not create this risk; it is a risk that is not directly related to research. The risks related to the research would include the risks of collecting blood and dust samples, conducting interviews, and so on.

## Regulations and Guidelines

U.S. federal regulations do not specifically address the observer’s dilemma, because they focus only on protecting human subjects from risks directly related to research. Federal regulations, for example, require that risks to human subjects be reasonable (in relation to benefits) and minimized. The regulations also require that investigators disclose reasonably foreseeable risks to the subjects [Department of Health and Human Services 2005; Food and Drug Administration (FDA) 2001; U.S. EPA 2006]. However, the risks covered by these regulations are generally understood to be risks directly related to research (FDA 1998; Wendler and Miller 2007). Other countries have research regulations similar to those adopted by the U.S. federal government (Amdur and Bankert 2005; Office of Human Research Protections 2009).

Agencies that provide interpretive guidance of the federal regulations, such as the Office of Human Research Protections and the Food and Drug Administration, also do not specifically address the observer’s dilemma. The Office of Human Research Protections’ informed consent guidance mentions that the institutional review board (IRB) may require investigators to provide subjects with any information that would help to protect their rights or welfare, but it does not give examples of what this information might consist of (Office of Human Research Protections 2008).

International ethical guidelines, such as the Nuremberg Code (1949), the Helsinki Declaration (World Medical Association 2008), and guidelines of the Council for International Organizations of Medical Sciences (2002), require that risks be reasonable, minimized, and disclosed, but again, these guidelines apply only to the risks directly related to research (Amdur and Bankert 2005). The Council for International Organizations of Medical Sciences (2002) guidelines require that investigators disclose all risks that a reasonable person would need to make a decision to participate in research, but they do not specify what these risks might include. The American Anthropological Association (1998) guidelines require investigators to avoid harming the safety, dignity, or privacy of research subjects, but they do not discuss investigators’ obligations to address risks not directly related to research.

Although research regulations and guidelines do not clearly state that investigators should protect human subjects from risks not directly related to research, there still remains the question of whether they have an ethical (or moral) obligation to do so. Responsible conduct of research involves more than just complying with laws and guidelines; one must also follow ethical standards and make ethical decisions (Emanuel et al. 2000).

## Ethical Obligations

To decide whether investigators have an ethical obligation to address risks not directly related to research, it will be useful to consider the conceptual framework found in the *Belmont Report* (National Commission 1979). Drafted by the National Commission for the Protection of Human Subjects of Biomedical and Behavioral Research, the *Belmont Report* served as a foundation for a major revision of the federal research regulations in 1981 and has had considerable influence over research with human subjects conducted in the United States and other countries (Shamoo and Resnik 2009). The *Belmont Report* is a useful source of guidance for investigators and IRBs because it articulates three principles—respect for persons, beneficence, and justice—that have broad support among ethical theories and cultural traditions.

Two of the *Belmont Report* principles support the idea that investigators have an ethical obligation to address risks not directly related to research. The principle of beneficence is a combination of two widely accepted moral maxims: do no harm and promote good. Beneficence requires investigators to minimize harms to research subjects and to maximize benefits to research subjects and society (National Commission 1979). One could argue that beneficence implies an obligation to address risks not directly related to research, because these risks can have a definite impact on subjects’ well-being. The principle of respect for persons obligates investigators to support the autonomous decision making of research subjects and provide additional protections for subjects with diminished autonomy. One could argue that this principle implies that investigators have an ethical obligation to inform subjects about risks not directly related to research, because this information would help to support the subjects’ autonomous decision making.

## Objections and Replies

Although two of the *Belmont Report* principles provide some support for an obligation to address risks not directly related to research, they do not yield conclusive proof for this ethical duty, because the principles are subject to interpretation. First, one could argue that investigators do not have an obligation to inform subjects about risks not directly related to their research if this information is not relevant to deciding whether to participate in a study. The investigator’s obligation to promote the subject’s autonomous decision making does not extend beyond choices related to research. Second, one could argue that addressing harms not directly related to research might have a positive impact on the subjects’ well-being but a negative impact on the population if addressing these risks undermines the subjects’ willingness to participate in the study, and thus research that could potentially benefit the population is not conducted. One could argue that as long as harms to subjects are minimized, investigators do not need to maximize benefits to subjects if this will adversely affect the research that is likely to benefit the population (Miller and Brody 2002).

Both of these objections to using the *Belmont Report* principles to support an obligation to address risks not related to research take a minimalist perspective on the investigator–subject relationship. According to this approach, investigators’ primary ethical duties are to conduct sound scientific research and to avoid harming or exploiting subjects (Miller and Brody 2002). It is unfortunate that research subjects encounter hazards in their natural environment, but because investigators did not create these risks, they are not responsible for them. Investigators are responsible only for minimizing the harms they cause, not extraneous harms—they are researchers, not rescuers. The best way to address the hazards that people face, according to this view, is to study these hazards carefully, so that knowledge-based interventions can be developed that may benefit the population.

A standard reply to these arguments against addressing risks not directly related to research is to assert that investigators should go beyond this minimalist ethic, because all people have positive duties to help others (Richardson and Belsky 2004). A variety of moral theories and traditions, including utilitarianism, Kantianism, virtue ethics, and Judeo–Christian ethics, assert that all people have an obligation to help others (Gert 2007). Consider, for example, the tragic case of Kitty Genovese, who was stabbed to death near her home in Queens, New York, on 13 March 1964. According to newspaper reports, 38 people saw her being stabbed or heard her screams but did nothing in response. No one even called the police (New York Times 1964). Most people would regard the behavior of the bystanders as appalling. The least that these witnesses could have done would be to pick up the phone to call the police. Likewise, investigators who are studying people in their environment should not always be neutral observers: Sometimes they should take action to help the people they are observing. Indeed, one might argue that investigators have special obligations to render aid over and above the obligations that all people have, given their knowledge and expertise—power implies responsibility (Richardson and Belsky 2004).

If one accepts the notion that investigators should go beyond the minimalist ethic and embrace a positive duty to help research subjects, many questions still need to be answered about how best to fulfill this obligation. Unlike prohibitions on murder, rape, or arson, beneficence is not an absolute rule that must always be followed (Gert 2007). Sometimes morality requires us to help others, and sometimes it does not, depending on the circumstances. For example, if a neighbor asks me to help him move furniture, but I have already promised to attend my son’s piano recital, then that prior obligation takes precedence over my obligation to help. In some circumstances, protecting one’s own life or health limits the duty to help others. For example, in the Kitty Genovese case, the bystanders did not have a moral obligation to risk their lives by thrusting themselves in the middle of the attack; calling the police would have been sufficient. Sometimes resource constraints require one to choose among different people to help. For example, medics on the battlefield often do not have enough time to help every wounded soldier at the same time, so they ration their time according to triage procedures (Repine et al. 2005). Finally, even when one has made a commitment to help, it can be difficult to decide exactly how to help. For example, there is a debate about whether it is better to send shipments of food to impoverished nations or to help develop their agriculture so that they can grow their own food (Sanchez 2009).

## Factors to Consider in Decision Making

These questions concerning the duty to render aid demonstrate that deciding whether and how to protect human subjects from risks not directly related to research is often not easy to do. The following are some considerations that can help investigators think through their obligations to protect human subjects from these risks.

### The evidence for the risks

Are the risks supported by solid evidence, or are they speculative? If there is considerable evidence for the risks, this would be a convincing reason for informing the subjects about the risks. The adverse effects of lead exposure on human health have been well studied. Lead can cause brain damage, learning disabilities, behavior problems, and stunted growth in children, and infertility, hypertension, joint pain, and memory problems in adults (U.S. EPA 2009). Investigators who are studying in-home exposures to lead should inform the subjects about unsafe lead levels that they detect (Resnik and Zeldin 2008). In the DDT example mentioned above, although the adverse effects of DDT on human health are not as well documented as the effects of lead, DDT does have some potential adverse effects on human health. The investigators could inform the subjects that DDT has some potential adverse effects on human health, but that these effects are still not well understood, and that they are conducting the study to learn more about them. Because DDT has had some adverse effects on pregnancy in laboratory animals, one of the main goals of their research is to answer questions about DDT’s effects on pregnancy in humans (Kegley et al. 2008).

### The nature of the risks

What are the possible adverse health effects of the environmental hazards? Do they cause significant harms, such as death, disability, or terminal illness, or only minor ones, such as headache, nausea, or dizziness? Are the harms permanent or temporary? Do they occur after brief exposures or only after prolonged exposures? What is a safe level of exposure for the environmental hazards? Asbestos, DDT, lead, arsenic, gamma radiation, and ozone are environmental hazards that have very different effects on the human body. Decisions to inform subjects about an environmental hazard should take into account the nature of the hazard and how it affects people.

### The usefulness of the information to the subjects

What can the subjects do with the information about risks? Is it useful to them? In the DDT case mentioned above, the information is potentially useful, because the subjects could take steps to minimize their exposure to DDT. Warning impoverished people living near a river about the risks of periodic flooding may not be very useful, because the people may already know about those risks, or they may not be able to take meaningful action in response to being warned, such as moving somewhere else.

### The effects on the study of informing the subjects

Are the subjects likely to change their behavior in a way that might affect the study? If the subjects are likely to change their behavior as a result of learning about hazards in their environment, it may be possible to design the study to take this change into account (Resnik and Wing 2007). In the DDT case, if some of the subjects stop using DDT around their home, they may still have some exposure to the chemical, because other villagers may be using DDT. When analyzing the data, the investigators could determine whether there is a relationship between the level of exposure and the outcome of interest (e.g., pregnancy loss). If there is no way to compensate for the subjects’ behavior, investigators will face a difficult choice: Should they inform the subjects, and perhaps jeopardize the validity of the study, or should they not inform the subjects, and fail to offer them some beneficial information?

### The effects on the community of informing the subjects

Will informing the subjects about environmental hazards help or harm the community? Very often, communicating risk information to the subjects will help the community. For example, if agricultural workers are informed about the risks of pesticide exposure, they may share this information with their peers, who may take steps to reduce their exposure, such as wearing more protective clothing. However, informing the subjects could sometimes have harmful effects on the community. In the DDT case, if the subjects are told that DDT is hazardous to human health, the villagers could stop using DDT to control mosquitoes that carry malaria, which could have an adverse impact on public health. Because the researchers have no evidence that DDT exposure is more dangerous than exposure to malaria-carrying mosquitoes, they should be mindful of how they discuss the hazards of DDT. They should communicate honestly and openly, guarding against alarming subjects unnecessarily.

## Conclusion

Deciding whether—and how—to inform research subjects about hazards they face in their environment can be a challenging task for investigators conducting observational research. Because legal rules and professional guidelines do not address this topic, investigators must carefully consider their ethical obligations in light of the facts and circumstances. To navigate through this dilemma, investigators should consider the evidence for the risks, the nature of the risks, the usefulness of risk information to the subjects, and the effects on the study and community of informing subjects about risks.
